# Combination Therapy of Ivabradine with Procainamide for the Management of Pediatric Postoperative Junctional Ectopic Tachycardia

**DOI:** 10.19102/icrm.2023.14075

**Published:** 2023-07-15

**Authors:** Soham Dasgupta, Christopher Johnsrude

**Affiliations:** ^1^Division of Pediatric Cardiology, Department of Pediatrics, Norton Children’s Hospital, University of Louisville School of Medicine, Louisville, KY, USA

**Keywords:** Ivabradine, junctional tachycardia, pediatrics, postoperative, procainamide

## Abstract

Pediatric postoperative junctional ectopic tachycardia (JET), although usually self-limited, may lead to significant morbidity and mortality. Anti-arrhythmic medications are often necessary to restore atrioventricular synchrony when non-pharmacological measures fail. Multiple drugs have been described for the management of postoperative JET, with enteral ivabradine being the latest addition. While safe administration of ivabradine has been described in combination with other anti-arrhythmics (amiodarone, flecainide), no study has described the use of ivabradine in conjunction with intravenous procainamide for the management of postoperative JET. Our case report describes the safe use of ivabradine and procainamide combination therapy in a young patient.

## Introduction

Pediatric junctional ectopic tachycardia (JET) after surgical repair of congenital heart defects occurs in 2%–10% of patients^1–4^ and can lead to low cardiac output secondary to short diastolic filling times and the loss of atrioventricular (AV) synchrony. If untreated, JET may result in significant morbidity and mortality, but is fortunately often self-limited and usually responds to interventions. Optimal management strategies aim to either restore sinus rhythm or control the ventricular rates to allow temporary atrial pacing at slightly faster rates. Most providers start with non-pharmacological interventions, including sedation with/without paralysis, core body cooling, optimization of serum electrolytes, and enhanced preload. Many patients require anti-arrhythmic agents (eg, intravenous amiodarone, procainamide, and sotalol),^5–9^ and drug selections vary by institutional protocols, preferences, and patient comorbidities.^10^

Ivabradine is a medication that has recently gained recognition for the safe and effective treatment of diverse tachyarrhythmias in adults; however, current pediatric literature is limited to case reports and small case series.^11^ There are several recent reports describing ivabradine in the treatment of postoperative JET.^12–14^ Arvind et al. concluded that oral ivabradine is not inferior to intravenous amiodarone in the conversion of postoperative JET to sinus rhythm.^15^ Others report ivabradine as a beneficial adjunct to other primary medications, including intravenous amiodarone, for postoperative and congenital JET.^13,16^ To date, there is no published experience reporting ivabradine use in patients receiving intravenous procainamide for postoperative JET. We describe the safe use of ivabradine and procainamide combination therapy.

## Case presentation

**Figure 1:**
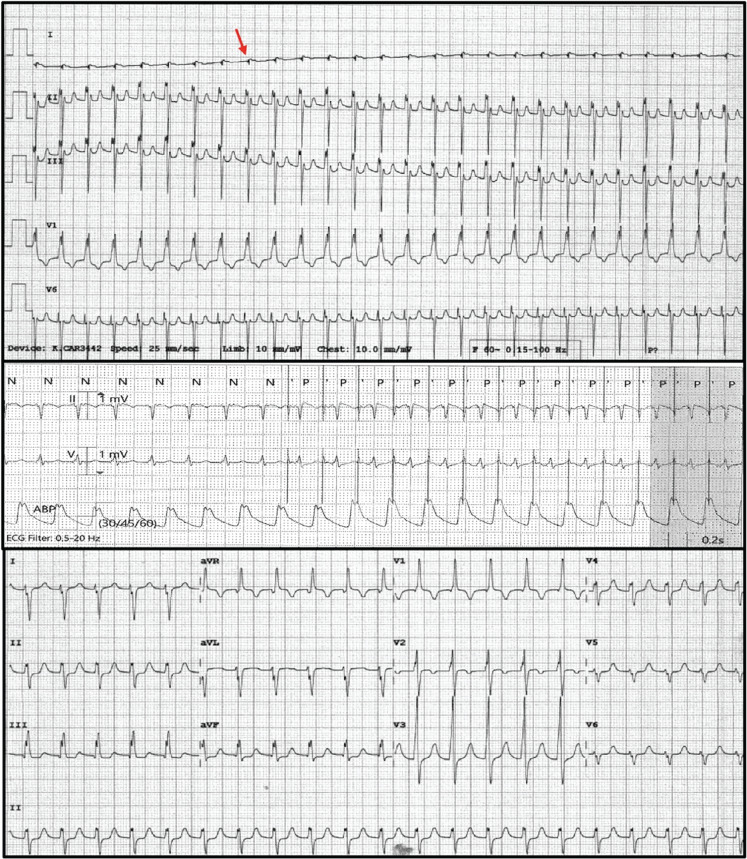
Atrial electrocardiograms (ECGs) of junctional ectopic tachycardia (JET). **A:** Atrial electrograms (red arrow) demonstrate simultaneous activation of the atria and ventricles consistent with 1:1 retrograde ventriculo-atrial conduction, as seen in some patients with JET. Connecting the atrial temporary pacing wires to the ECG machine affected the QRS axis in the frontal plane. **B:** A telemetry strip demonstrates a slower junctional rate after ivabradine administration, allowing atrial pacing above the junctional rate, restoring atrioventricular synchrony and clearly improving arterial blood pressure. **C:** A 12-lead ECG demonstrates a markedly slower JET (accelerated junctional rhythm) after administration of enteral ivabradine.

A 5-year-old girl with hypoplastic left heart syndrome underwent an extracardiac non-fenestrated Fontan operation. She was in sinus rhythm when she required emergent surgical Fontan fenestration for hypotension and low cardiac output on postoperative day (POD) #0, and subsequently returned to the cardiac intensive care unit on a venoarterial extracorporeal membrane oxygenator (ECMO). The patient had a pair of atrial and a pair of ventricular temporary epicardial pacing wires, respectively, and received intravenous dexmedetomidine (minimum dose, 0.5 μg/kg/min) in the immediate postoperative period.^17^ On POD #1, she developed hemodynamically significant JET to 170 bpm, which was diagnosed based on narrow QRS complexes similar to sinus rhythm, with warm-up/cool-down behavior and expected responses to therapeutic interventions (described below)^18^ **(Figure 1)**. Once confirmed, our standard JET management protocol was initiated. The patient was sedated and paralyzed, and her core temperature was cooled to 34°C–35°C. Serum electrolytes, including calcium, magnesium, and potassium, were normalized, and intravenous catecholamine support was reduced as tolerated. Intravenous procainamide was administered as a 5 mg/kg bolus × 2, followed by infusion at 20 μg/kg/min. Serial bloodwork demonstrated rising hepatic transaminases (aspartate transaminase, 402 U/L; alanine transaminase, 1173 U/L) and evidence of acute kidney injury (serum creatinine, 1.75 mg/dL), and JET persisted despite increasing the procainamide infusion to 35 μg/kg/min. The procainamide/N-acetylprocainamide (NAPA) levels were 4.7 and 3.4 μg/mL, respectively, at this time (therapeutic procainamide level, 4–10 μg/mL; no specific range available for NAPA). Enteral ivabradine was added via a nasogastric tube (0.05 mg/kg/dose every 12 h). Within 4 h of the first dose, the JET rate fell to 130–140 bpm, and slightly faster atrial pacing restored AV synchrony **(Figure 1)**. The patient underwent ECMO decannulation uneventfully the following day, and both procainamide and ivabradine were discontinued the next day, with no further recurrence of JET. We did not note any adverse electrocardiogram (ECG), laboratory marker, or hemodynamic effects when using the combination of procainamide and ivabradine.

Institutional review board approval was obtained prior to the preparation of this manuscript.

## Discussion

Postoperative JET is an important arrhythmia that can occur after surgery for congenital heart disease, and it appears to reflect abnormal phase 4 automaticity originating in the AV node or bundle of His.^19^ JET occurs in 2%–10% of patients, and the incidence is influenced by patient age, underlying congenital heart disease, type/technique of cardiac surgery, and other perioperative factors.^2,3,20,21^ JET may be caused by direct mechanical trauma or indirect stretch injury of these tissues or by insufficient myocardial preservation.^19^ JET usually manifests soon after the cessation of bypass and, if rapid and untreated, can cause significant hemodynamic embarrassment. Fortunately, JET is most often transient, resolving within 72 h.^2^

While many patients respond to non-pharmacological therapy, JET sometimes persists and progresses, necessitating administration of anti-arrhythmic drugs.^22^ Recently, enteral ivabradine administered alone or in combination with other medications (amiodarone, flecainide) has been shown to be effective.^13,16,23^ To date, there have been no reports using ivabradine in combination with procainamide for the management of postoperative JET. We describe a patient with JET refractory to initial measures with a progressively worsening postoperative status, in whom the addition of ivabradine to procainamide proved effective and safe.

The two anti-arrhythmics used most often to treat postoperative JET are intravenous amiodarone and intravenous procainamide.^6,8^ The initial choice between these is often guided by institutional and provider preference and the ability to regularly monitor serum procainamide levels. Despite the conversation of JET to sinus rhythm in 45%–86% of patients,^5–7^ intravenous amiodarone may be associated with significant adverse effects in fragile postoperative pediatric patients, potentially contributing to significant hypotension, bradycardia, and AV block.^7,24^ Procainamide is a class 1A anti-arrhythmic with demonstrated efficacy in the management of JET by suppressing or slowing JET without significant hypotension or myocardial depression—a particular concern in patients fresh from cardiac surgery.^25–27^ Appropriate use of procainamide requires following the serial procainamide/NAPA levels to influence dosing and to monitor for toxicity; procainamide is generally avoided in patients with significant liver or renal dysfunction.^28^ Recent reports have provided limited but growing experience showing that ivabradine can be effective and safe in the management of various tachyarrhythmias, including JET. Ivabradine can slow or terminate tachyarrhythmias by blocking hyperpolarization-activated cyclic nucleotide-gated transmembrane sodium channels, thereby inhibiting an abnormally overactive *I*_f_ current.^13^ A recent study demonstrated that enteral ivabradine is not inferior to intravenous amiodarone in the conversion of postoperative JET to sinus rhythm; the authors concluded that monotherapy with ivabradine may be considered as an alternative to amiodarone for some of these patients.^15^ Additionally, prior case studies have described the benefits of amiodarone or flecainide in conjunction with ivabradine for the treatment of postoperative and congenital JET.^16,23^

While conversion to sinus rhythm may be expected to take longer with enteral versus intravenous agents, our case and prior reports demonstrate that rate control of JET can be achieved fairly quickly with ivabradine (within a few hours), slowing JET to permit temporary atrial pacing and restore AV synchrony.^4,14,15^ Furthermore, despite concern that enteral absorption and effectiveness of a medication such as ivabradine in a critically ill patient on ECMO may be delayed or blunted, our experience and other reports indicate that ivabradine at standard dosing can be very successful.^4,15^ Of note, modern ECMO circuits do not appear to significantly impact drug pharmacodynamics compared to the critical illness itself.^29^ In addition, our patient did not develop bradycardia, hypotension, or worrisome ECG changes, extending the observation that ivabradine in these critically ill patients appears to be safe.^13,16,23^

Therefore, if JET proves refractory to initial treatment with intravenous procainamide, significant benefit may result from early addition of ivabradine before escalating the procainamide infusion or changing anti-arrhythmic therapy to intravenous amiodarone due to its potential adverse effects.

## Conclusion

Management of postoperative JET can be challenging, and monotherapy or combination anti-arrhythmic therapy is sometimes necessary to slow the rate and facilitate restoration of AV synchrony with temporary atrial pacing. Ivabradine administered alone or in combination with other anti-arrhythmics appears to be an important and safe treatment option. At our institution, intravenous procainamide is generally the initial medication used in these patients, and we report the safe and effective addition of ivabradine to procainamide in a critically ill child. Larger studies at other institutions are necessary to confirm the impact of procainamide/ivabradine combination therapy for postoperative JET.
